# Our Homes

**Published:** 1880-05

**Authors:** 


OUR HOMES. By Henry Hartshorn, A.M., M.D., formerly Professor of Hygiene in the University of Pennsylvania. Philadelphia: PRESLEY BLAKISTON, 1012 Walnut street. 1880.

